# The enforcement of statewide mask wearing mandates to prevent COVID-19 in the US: an overview

**DOI:** 10.12688/f1000research.25907.1

**Published:** 2020-09-07

**Authors:** Philip Jacobs, Arvi P. Ohinmaa

**Affiliations:** 1 Department of Medicine, University of Alberta, Edmonton, Alberta, T5T 2W8, Canada

**Keywords:** COVID-19, face masks, statewide mandates, governor's order, enforcement

## Abstract

Face masks have become the bulwark of COVID-19 prevention in the US.  Between 10 April and 1 August, 2020, 33 state governors issued orders requiring businesses to require their customers and employees to wear face masks, and persons outdoors who could not social distance  to do the same. We documented the policies and enforcement actions for these policies in each of the states.  We used governors’ orders and journalists’ news reports as our sources. Our results show that the states used a variety of state and local (county and municipality) agencies to enforce business prevention behaviors and primarily local  law enforcement agencies to enforce outside mask-wearing behaviours. In particular, law enforcement officers demonstrated a strong preference for educating non-mask wearers, and indicated a reluctance to resort to civil penalties that were enacted in the state orders.  Businesses expressed a preference to have government agencies enforce non-mask wearing behaviours.  But there was also a widespread reluctance on the part of local law enforcement  to resort to legal remedies.

## Introduction

In the United States, the Centers for Disease Control first recommended the wearing of face masks on April 3, 2020. On April 8, The Governor of New Jersey was the first to issue a general, statewide mandate, which required “workers and customers to wear cloth face coverings while on the premises”. By August 1, 33 state governors had issued state-wide mask wearing orders. The resulting pattern of regulations regarding mask wearing across the states is a welter. There is wide variation in the design of the ordinances, including whose behavior is being targeted, what is expected of them, when they must observe the ordinances, where their behavior is to be regulated, why they must submit to these orders, and how violations will be enforced. In this paper we summarize the introduction of state policies in those states which issued statewide mandates, and how these mandates are being enforced.

## Methods

Our subjects were the 33 states which had mask mandates in effect by August 1.

We used newspaper and broadcasting articles as our primary sources. We identified these articles using Google searches ending August 4 with the following keywords: ”COVID-19”, “State” (e.g., “Pennsylvania”) AND “mask order” or “mask mandate” AND “enforcement” OR “education”. We used newspaper or broadcast articles as the primary source because they contained essential information about the mandates, as well as additional information about enforcement. We identify primary sources for each state in the *Underlying data*, Appendix 1 ^1^. Most news sources came from local broadcast stations, local and national news services, networks including the Public Broadcasting System, CBS, and ABC. We used the actual ordinances as secondary sources, but these often did not contain information about enforcement.

We abstracted the following items for each state:

**Are businesses given first-line responsibility for their customers and employees?** By reading news articles, we determined if businesses were directly responsible for mask wearing behaviors of customers and employees on their premises. Answers were “Yes,” “No,” or in a few cases, “Unsure.”

**What authorities enforce whether businesses apply mask wearing orders on their premises?** We determined from the news articles which government agencies were responsible for ensuring that businesses were applying the governors’ orders. This information might also be obtained from the governors’ orders. For each state, we listed these authorities: “LLE” indicates “Local Law enforcement,” “LPH” signifies “Local Public Health,” and “DOH” signifies “Department of Health.”

**What authorities enforce mask wearing behaviors outside of business premises?** We identified the enforcement authorities from the news articles. Abbreviations are the same as the previous question.

**Is education viewed as a primary tool to encourage mask wearing behaviors?** We obtained this information from news articles. Answers were “Yes” or “No”.

**Do local governments (counties and municipalities) pass their own orders?** We obtained this information by searching news articles which identified additional county or municipal mask orders within individual states. Answers were “Yes” or “No”.

**Has local law enforcement shown any resistance to enforcing statewide orders?** We obtained this information by searching for news articles that identified local law enforcers’ comments on “enforcement.” Answers were “Yes” or “No”.

## Results

The publications on which the analysis was based are shown in the *Underlying data*, Appendix 1 ^1^. These data underlying the analysis are shown in the Data Availability section.

### Which states issued statewide mandates?

**Figure 1.**
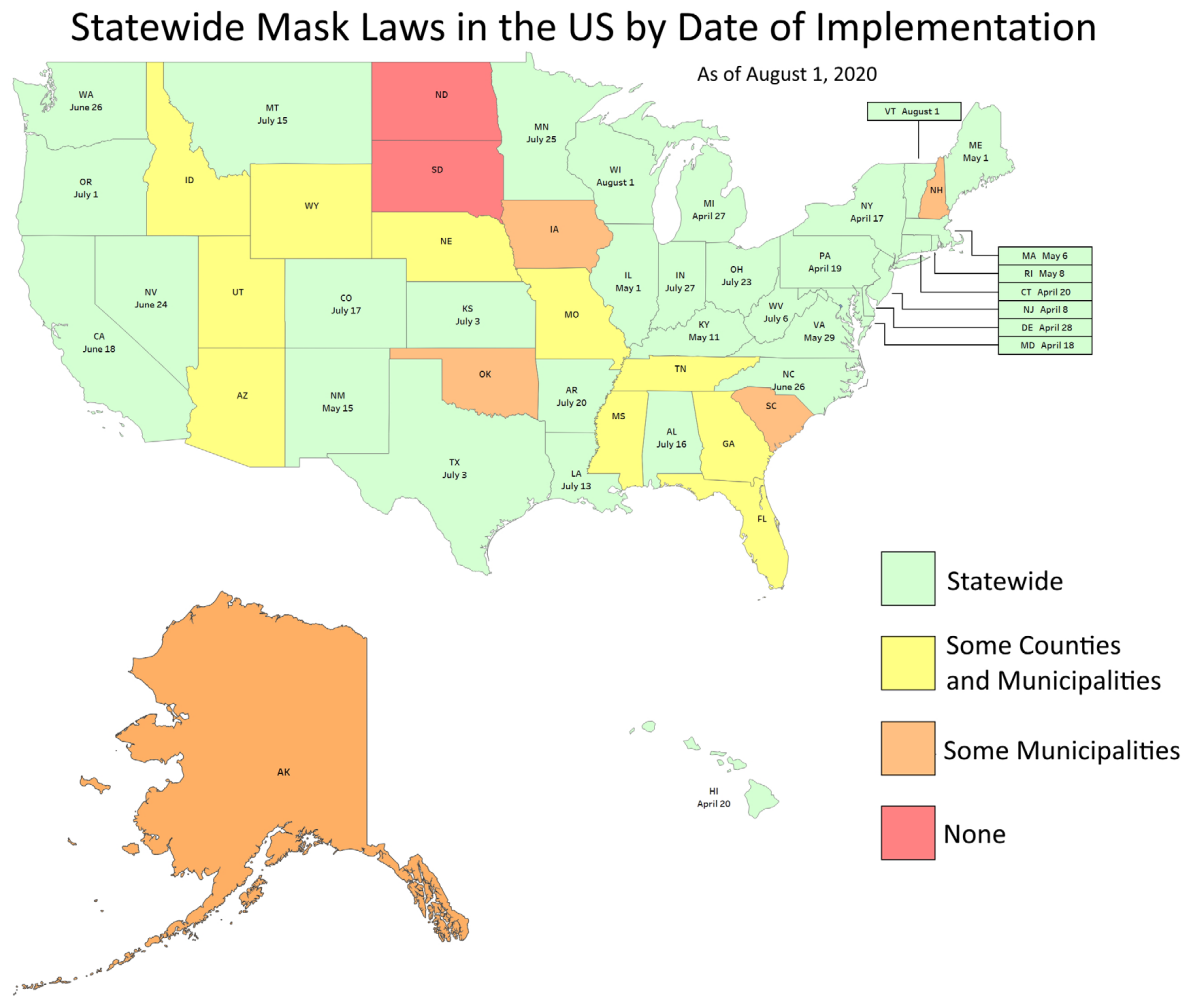
In effect dates of statewide orders by US state. The first state order (New Jersey) came in effect on April 8, 2020. We followed trends until 1 August, 2020. State acronyms are as follows. Alabama (AL), Alaska (AK), Arizona (AZ), Arkansas (AR), California (CA), Colorado (CO), Connecticut (CT), Delaware (DE), Florida (FL), Georgia (GA), Hawaii (HI), Idaho (ID), Illinois (IL), Indiana (IN), Iowa (IA), Kansas (KS), Kentucky (KY), Louisiana (LA), Maine (ME), Maryland (MD), Massachusetts (MA), Michigan (MI), Minnesota (MN), Mississippi (MS), Missouri (MO), Montana (MT), Nebraska (NE), Nevada (NV), New Hampshire (NH), New Jersey (NJ), New Mexico (NM), New York (NY), North Carolina (NC), North Dakota (ND), Ohio (OH), Oklahoma (OK), Oregon (OR), Pennsylvania (PA), Rhode Island (RI), South Carolina (SC), South Dakota (SD), Tennessee (TN), Texas (TX), Utah (UT), Vermont (VT), Virginia (VA), Washington (WA), West Virginia (WV), Wisconsin (WI), Wyoming (WY).

We identify the 33 states with statewide mandates (green) in Figure 1. We did not cover states with county or municipal orders (yellow), states with only municipal orders (brown) or states with no orders (red). 

### Who was required to wear masks?

The entire population, with exceptions, was included in the order. Persons exempted from the orders were persons with disabilities or medical conditions who were over specific ages. The age above which masks were mandatory were 2 (14 states), 4 (3 states), 5 (3 states), 6 (1 state), 7 (2 states), 9 (4 states), 10 (4 states), 11 (1 state) and 12 (1 state). See Table 1.

**Table 1.** Data for US states with statewide mask orders. Includes date the order was in effect, fines and sentences, and maximum age for exemption.

State	Date of Mask Order	Penalty	Exceptions (Age)
Alabama	16-Jul-20	$500 fine and/or jail	Aged 6 or younger
Arkansas	20-Jul-20	$100-500 fine	Aged 10 or younger
California	18-Jun-20	Misdemeanor + fine	Aged 2 or younger
Colorado	17-Jul-20	"Civil or criminal penalties"	Aged 10 or younger
Connecticut	20-Apr-20		Aged 2 or younger
Delaware	28-Apr-20	Fine up to $500, jail up to 6 months	Aged 12 or younger
Hawaii	20-Apr-20	Fine up to $5000, jail up to 1 year	Aged 4 or younger
Illinois	01-May-20	Varies by locality	Aged 2 or younger
Indiana	27-Jul-20	Planned penalties removed	Aged 7 or younger
Kansas	03-Jul-20	Varies by locality	Aged 5 or younger
Kentucky	11-May-20	Fine (starting July 10)	Aged 4 or younger
Louisiana	13-Jul-20	Varies by locality	Aged 7 or younger
Maine	01-May-20	Varies by locality	Aged 2 or younger
Maryland	18-Apr-20	Fine up to $5000, jail up to 1 year	Aged 2 or younger
Massachusetts	06-May-20	Fine up to $300	Aged 2 or younger
Michigan	27-Apr-20	Fine up to $500 (starting July 13)	Aged 2 or younger
Minnesota	25-Jul-20	Fine up to $100	Aged 5 or younger
Montana	15-Jul-20	Trespassing charge	Aged 4 or younger
Nevada	24-Jun-20		Aged 10 or younger
New Jersey	08-Apr-20	Fine up to $1000, jail up to 6 months (starting July 8)	Aged 2 or younger
New Mexico	15-May-20	Fine up to $100 (starting July 1)	Aged 2 or younger
New York	17-Apr-20	Fine up to $1000 (Starting July 9)	Aged 2 or younger
North Carolina	26-Jun-20	Trespassing charge	Aged 10 or younger
Ohio	23-Jul-20	Fine up to $750, jail up to 30 days	Aged 9 or younger
Oregon	01-Jul-20	Fine up to $1250, jail up to 30 days (starting July 13)	Aged 11 or younger
Pennsylvania	19-Apr-20		Aged 2 or younger
Rhode Island	08-May-20	Varies by locality	Aged 2 or younger
Texas	03-Jul-20	Fine up to $250 (repeat offenders)	Aged 9 or younger
Vermont	01-Aug-20		Aged under 2
Virginia	29-May-20	Fine up to $2500, jail up to 1 year	Aged 9 or younger
Washington	26-Jun-20	Fine up to $1000, jail up to 90 days	Aged 2 or younger
West Virginia	06-Jul-20		Aged 9 or younger
Wisconsin	01-Aug-20	Fine up to $200	Aged 5 or younger

### When did the mandate become effective?

Mandates became effective in the months of April (8 states), May (7 states), June (4 states), July (12 states) and August (2 states). See Figure 1.

### What was the scope of the mandate (indoor, indoor and outdoor, or “in public”)?

The mandates covered indoor only (8 states: Colorado, Hawaii, Indiana, Maine, Montana, Minnesota, Montana, Oregon, and West Virginia), outdoor and indoor or “public” places usually where recommended spacing was not available or there were large crowds (25 states). Some states excluded certain industries (e.g., gyms) if they did not meet public health requirements. See Table 2 for individual state information.

**Table 2.** Enforcement of statewide mask orders. Enforcement includes business responsibility, the state authority that enforces both inside and outdoor subjects, whether states emphasize education regarding mask wearing, whether local governments also impose mask orders, and if local law enforcement resisted a role in enforcing the order. L=local, LE = law enforcement, PH=public health, OSH=occupational safety and health, DOH=department of health.

State	Business responsible for customers / employers?	Authority that enforces businesses	Authority that enforces outdoor mask wearers	Education a primary prevention tool?	Local governments may also pass own orders	Resistance from Local Law Enforcement
**Alabama**	Yes	No	LLE	Yes	No	Yes
**Arkansas**	No	None	LLE	Yes	Yes	Yes
**California**	Yes	Alc.Bev. Control	LLE	Yes	Yes	Yes
**Colorado**	Yes	Agencies like state liq. Control board	PH, LLE	Yes	Yes	Yes
**Connecticut**	Yes	PH & LLE	LLE	Yes	No	No
**Delaware**	Yes	PH	PH	Yes		No
**Hawaii**	Yes	LLE	LLE	No	Yes	Yes
**Illinois**	Yes	None	LLE	Yes	Yes	No
**Indiana**	No	State & local PH	State & L PH	Yes		No
**Kansas**	Yes	LLE	LLE	Yes	Yes	Yes
**Kentucky**	Yes	LPH & Labor Cabinet	LLE	No	No	Yes
**Louisiana**	Yes	state fire marshall	LLE	No	Yes	Yes
**Maine**	Yes	Business regulators			Yes	
**Massachusetts**	Unsure	Unsure	LLE	Yes		No
**Michigan**	Yes	state licensing body	LLE	No	No	Yes
**Minnesota**	Yes	LLF				Yes
**Montana**	Yes	LLE, LPH	LLE	Yes	No	Yes
**Nevada**	Yes	OSHA	Limited	Yes	No	Yes
**New Jersey**			LLE	Yes		Yes
**New Mexico**	Yes	State pol, DOH	Limited	Yes	No	Yes
**New York**	Yes	State/cty DOH	LLE	Yes	Yes	Yes
**North Carolina**	Yes	LLE	LLE	Yes	No	Yes
**Ohio**	Yes	LHD / OH Invest. Unit	Unclear	Yes	No	Yes
**Oregon**	Yes	OSH	LLE	Yes	No	Yes
**Pennsylvania**	Unsure	Not enforced	LLE	Yes	No	Yes
**Rhode Island**	Yes	RI Dept Bus Reg	LLE	Yes	No	Tes
**Texas**	Yes	Local govt	LLE	Yes	Yes	Yes
**Vermont**	Yes	Voluntary	None	Yes	No	No
**Virginia**	Yes	DOH, state licensing agencies	Focus on business	Yes	No	Yes
**Washington**	Yes	WA Dept of Labor & Industries	Unclear	Yes	No	Yes
**West Virginia**	Yes	Business self- enforcement	None	Yes	No	No
**Wisconsin**	Uncertain	PH	Local and state officials	Yes	No	Yes

### Who was the primary target of the violation: businesses or individual non-mask wearing violators?

In 26 states, businesses and establishments formed the primary targeted group. Three states (Arkansas, Indiana, New Jersey) targeted only individual mask non-wearers. In two states (Pennsylvania, Wisconsin) we were unsure of the targeted group. See Table 2.

### What is the maximum penalty of a violation?

Ten states had provisions for fines only. The usual amount was between $100 and $200, but NY had a maximum of $1,000. Nine states had provisions for fines and/or jail sentences. The maximum fine in this group was $5,000 (Hawaii, Maryland) and the maximum jail sentence was one year (Maryland), but these amounts were outliers. More typical values were $500 fines and/or 30 days to six months in jail. In five states fines varied by county. The other states did not specify penalties, and some of these could have no penalty: in a number of states sheriffs expressed a strong preference for education over criminal or even civil proceedings. See Table 2 for individual state information.

### Who is the enforcement agency?

In most of the states (27 states) the government relied on private businesses to enforce mask wearing behaviors. Three states (Arkansas, Indiana, New Jersey) focused directly on non-mask-wearers. We were unsure of the governors’ focuses in three other states (Massachusetts, Pennsylvania, Wisconsin). In addition, because many states extended the mandate to outdoor non-business settings, there was enforcement in these settings as well. The pattern of enforcement in the two settings was very different.

Agencies which enforced businesses include state occupation safety and health agencies (Nevada, Ohio, Oregon, Washington, Kentucky); state or local public health agencies (Conneticut, Delaware, Indiana, Kentucky, Montana, New Mexico, New York, Ohio, Virginia, Wisconsin), city or county law enforcement (Connecticut, Hawaii, Kansas, North Carolina), alcohol and beverage control (California, Colorado), business regulators and licensers (Maine, Mississippi, Rhode Island). In some cases regulators were not specified, or the area was not enforced by the government. The enforcement of non-mask wearing behavior *outside of businesses* was usually assigned to local law enforcement (19 states) or public health (3 states). In other cases, there was limited to no enforcement outside the business setting. Many local sheriffs or local police chiefs stated their objections to enforcement on grounds that there were limited resources; a few also mentioned constitutional grounds.

## Discussion/conclusions

Based on public and journalist reports, we analyzed the introduction and enforcement of statewide mask wearing mandates in the 33 US states that imposed such orders between April 10 and August 1, 2020. Most states relied on businesses to enforce customer and employee mask wearing behavior. However, both the contents of the governors’ orders and the type and degree of enforcement varied widely between the states. Enforcement responsibility for personal (outdoor) mask wearing behavior was handed over to local law enforcement.

The business sector of the economy has been very active in COVID-19 prevention during this time period by introducing mask wearing regulations for its customers and employees. A survey of large US retail chains showed that 16 chains had introduced cross-country mask wearing policies in May, 2 in June, and 34 in July. Despite the adoption of storewide prevention policies, businesses, their trade associations, and employee trade unions have expressed concern at taking on primary enforcement roles. Private companies still relied on state laws to provide them with a rationale for requiring customers to wear masks. These businesses are subject to degrees of enforcement that have been inconsistent across states and that have often been lax. Many governors’ orders also covered outdoor areas, and most of these were nominally enforced by local law enforcement agencies. However, senior law enforcement officers in all states issued statements that they would not enforce mask wearing orders. For example, 38 sheriffs in Montana issued an op-ed which stated that a mask wearing directive “is not a mandate for law enforcement to issue citations and arrest violators.” Although such statements were not universal, examples can be found in every state.

We used both governors’ orders and news reports for our data sources. Policies like engaging in public education are subjectively described and data for them are not collected; instead we used sheriffs’ interviews with journalists to document the policies. Also, we could not obtain data on citations written by local law enforcement. Nevertheless, information on education and enforcement was widely reported in the press across the nation, and strongly suggests a national trend. It also indicates that mask wearing behavior, now considered a bulwark against the spread of COVID-19, is not being strongly enforced, especially in the outdoor sectors. 

## Data availability

Harvard Dataverse: Press articles on statewide mask orders in 33 US states. https://doi.org/10.7939/DVN/SFIT0R ^1^

This project contains the following underlying data:

- **Appendix 1 Press Publications.** This file contains the news articles that we used to abstract the descriptive variables. It includes state, topic, and internet address.
