# Supplementary material for: The enforcement of statewide mask wearing mandates to prevent COVID-19 in the US: an overview
Source: F1000Res. 2020 Sep 7;9:1100. [Version 1] doi: 10.12688/f1000research.25907.1 (PMC7818140; doi:10.12688/f1000research.25907.1)
Supplement: Peer review history

# Peer review: Reviewer response for version 1

**DOI:** 10.5256/f1000research.28591.r75987

**Published:** 2021-01-20

**Authors:** Charles Hunt, Hina Shah

**Affiliations:** 1 Kansas Health Institute, Topeka, KS, USA

This is a descriptive study of state-level mask mandates in the U.S. that were implemented between April 10, 2020, and August 1, 2020, to mitigate the spread of COVID-19. The objective of the study is to document which states had issued mask mandates, to characterize the mask mandates and to assess how the mandates were being enforced. The study also assessed whether law enforcement officials had demonstrated resistance to enforcing mask mandates.

 The study methodology involved conducting online searches using Google for newspaper and broadcast articles as the primary data source. The actual text of the orders (described as “ordinances”) was identified as a secondary data source. The documents were reviewed, and specific characteristics of interest were abstracted from the text.

 The study found that policies varied substantially among the 33 states that had issued mask mandates, particularly with respect to enforcement and penalty provisions. State policies varied also by authority of local governments to issue mask mandates and exceptions (e.g., age, medical contraindications). While the degree to which law enforcement officials had demonstrated resistance to enforcement was not characterized in the study, the authors noted that examples of resistance could be found in every state.

 The article clearly describes the methodology used to identify which states had mask mandates, including the specific online search terms and other parameters. While general descriptions of the abstract categories were provided (e.g., enforcement requirements for businesses, which authorities enforce business requirements, whether education is viewed as a primary tool, etc.), the article did not include detailed definitions for each category. The article also did not describe how the actual text of the orders was utilized in the abstraction process. These methodology details might provide additional clarity with respect to how the states were categorized. For example, Kansas is categorized as having a statewide mask law. While that is correct, the law also allows local boards of county commissioners to adopt orders that are less stringent than the state order, thereby giving counties the option to opt out, which many have done ^1^, ^2^. The article provides a link to the source data in the form of a Microsoft Word document with URLs for each article or order by state.

 Reproducibility of the study is uncertain given the qualitative nature of the methods, the lack of coding method details and reliance on news articles as the primary data source. In addition, while URLs are provided for each source document, full citation details are not provided. As a result, links might be obsolete, or the content might have changed. In our own government mitigation policy research, we have found it challenging to interpret and consistently categorize aspects of public health orders and other official governmental mitigation policy actions. In addition, we have often found discrepancies between published news reports and the actual text of the orders. Nonetheless, the article is clearly written and well-organized, the conclusions appear sound, and the study makes an important contribution to understanding the challenges of implementing and enforcing population-level policies to control the spread of COVID-19.

 (1) Kansas Health Institute. A Kansas Twist—Reopening plans for Kansas counties. Available https://www.khi.org/policy/article/20-25 ^1^

 (2) Van Dyke ME, Rogers TM, Pevzner E, Satterwhite CL, Shah HB, Beckman WJ, Ahmed F, Hunt DC, Rule J. Trends in county-level COVID-19 incidence in counties with and without a mask mandate – Kansas, June 1 – August 23 23, 2020. *Morbidity and Mortality Weekly Report*, 2020, 69(47), 1777-1781. ^2^

Is the work clearly and accurately presented and does it cite the current literature?

Yes

If applicable, is the statistical analysis and its interpretation appropriate?

Not applicable

Are all the source data underlying the results available to ensure full reproducibility?

Partly

Is the study design appropriate and is the work technically sound?

Yes

Are the conclusions drawn adequately supported by the results?

Yes

Are sufficient details of methods and analysis provided to allow replication by others?

Partly

Reviewer Expertise:

Epidemiology; public health systems; public health policy

We confirm that we have read this submission and believe that we have an appropriate level of expertise to confirm that it is of an acceptable scientific standard.

## References

[1] A Kansas Twist—Reopening plans for Kansas counties. *Kansas Health Institute*. Reference source

[2] : Trends in county-level COVID-19 incidence in counties with and without a mask mandate. *Morbidity and Mortality Weekly Report*.2020;10.15585/mmwr.mm6947e2PMC772760533237889

# Peer review: Reviewer response for version 1

**DOI:** 10.5256/f1000research.28591.r75986

**Published:** 2020-12-10

**Authors:** Evelyn Forget

**Affiliations:** 1 Rady Faculty of Health Sciences, University of Manitoba, Winnipeg, MB, Canada

This article documents policies and enforcement actions in the 33 states that adopted face mask policies between 10 April and 1 August 2020, by examining news articles and broadcasting articles retrieved through Google on August 4. They found that states used a variety of state and local agencies to enforce policies, and that these agencies overwhelmingly focused on education rather than legal penalties.

 The article is descriptive in nature. The methods are straightforward and clearly articulated, including search terms, databases and dates of access. The methodology is appropriate. The author has made the data retrieved available and therefore it is possible to replicate the study, reproduce it in the future to determine whether enforcement agencies or policies have changed over time as case positivity rates, vaccination availability and/or mortality change, and to augment the study by using additional search terms on the original or alternative databases.

 The article is clearly written, and the conclusions supported by the evidence.

Is the work clearly and accurately presented and does it cite the current literature?

Yes

If applicable, is the statistical analysis and its interpretation appropriate?

Not applicable

Are all the source data underlying the results available to ensure full reproducibility?

Yes

Is the study design appropriate and is the work technically sound?

Yes

Are the conclusions drawn adequately supported by the results?

Yes

Are sufficient details of methods and analysis provided to allow replication by others?

Yes

Reviewer Expertise:

Health economics; health policy

I confirm that I have read this submission and believe that I have an appropriate level of expertise to confirm that it is of an acceptable scientific standard.
